# Implementation of a soft grading system for chemistry in a Moodle plugin

**DOI:** 10.1186/s13321-022-00645-0

**Published:** 2022-10-26

**Authors:** Louis Plyer, Gilles Marcou, Céline Perves, Rachel Schurhammer, Alexandre Varnek

**Affiliations:** 1 Faculté de Chimie, University of Strasbourg, Strasbourg, France; 2 Laboratory of Chemoinformatics–UMR7140, University of Strasbourg, Strasbourg, France; 3 Direction du Numérique (DNUM), University of Strasbourg, Strasbourg, France

**Keywords:** Educational chemistry, Softgrading, Moodle, Plugin

## Abstract

We report a novel approach for grading chemical structure drawings for remote teaching, integrated into the Moodle platform. Typically, existing online platforms use a binary grading system, which often fails to give a nuanced evaluation of the answers given by the students. Therefore, such platforms are unevenly adapted to different disciplines. This is particularly true in the case of chemical structures, where most questions simply cannot be evaluated on a true/false basis. Specifically, a strict comparison of candidate and expected chemical structures is not sufficient when some tolerance is deemed acceptable. To overcome this limitation, we have developed a grading workflow based on the pairwise similarity score of two considered chemical structures. This workflow is implemented as a Moodle plugin, using the Chemdoodle engine for drawing structures and communicating with a REST server to compute the similarity score using molecular descriptors. The plugin (https://github.com/Laboratoire-de-Chemoinformatique/moodle-qtype_molsimilarity) is easily adaptable to any academic user; both embedding and similarity measures can be configured.

## Introduction

Several solutions have been proposed in the past few years for the remote teaching of chemistry. One of the first tools implying using a chemical structure sketcher for organic chemistry online tutorials with automated correction was described by O'Sullivan and Hargarden [1]. The drawing prepared by the student is exported to a canonical SMILES (Simplified Molecular Input Line Entry Specification) [2] string, followed by the evaluation based on its comparison with an expected answer in SMILES format. Such a solution is realized in the SOCOT platform maintained by the University of Cork and the Dublin Institute of Technology. A similar solution was developed by Flynn et al*.* [3] for learning nomenclature in chemistry; it is accessible on the *nomenclature101.com* web service hosted by the University of Ottawa. Morsh and Lewis [4] described how the teacher and the students exchange chemistry questions and answers at the University of Illinois–Springfield and at Saint-Louis University, using a touchpad.

OpenOChem [5] is another tool accessible from several Learning Management Systems (LMS). Therefore, it leaves room for Learning Tools Integration (LTI). Unfortunately, the solutions described in [4, 5] cannot be integrated with Moodle [6] or Scenari [7]—Scenari is a popular LMS in France. Moreover, these solutions are based on the ChemAxon web services [8], which are free for academic organizations as long as the company maintains this policy.

Most existing online platforms use a binary grading system, implying a strict comparison of the two canonical SMILES. However, as noticed by Richards-Babb et al*.* [9], questions whose solutions are based on a limited number of choices are often ineffective for self-assessment. According to their estimations, about a third of students simply try different suggested choices instead of turning to the course or remediation materials when they do not know the answer. It should also be noted that some questions need smooth grading. A typical example concerns the demand to prepare a chemical structure corresponding to a given SMILES string. The binary assessment results in a grade equal to zero in case of any minor error, whereas the smooth assessment distinguishes the level of students as a function of the number of mistakes in the answer.

Here, we describe a novel software tool able to perform a smooth grading of chemical structures using the Moodle platform. In order to make the tool accessible to any educational institution, we follow the Moodle philosophy [10], so the plugin and all its components are free and open source. Unlike already existing plugins, our tool doesn’t transform chemical structures into canonical SMILES because the latter can hardly be applied in certain chemistry case studies [11]. Instead, InChI strings and ISIDA fragment descriptors were used for chemical structure encoding, whereas a pairwise Tanimoto similarity score for teacher/student structures was used for a smooth evaluation of students.

### Development

**Fig. 1:**
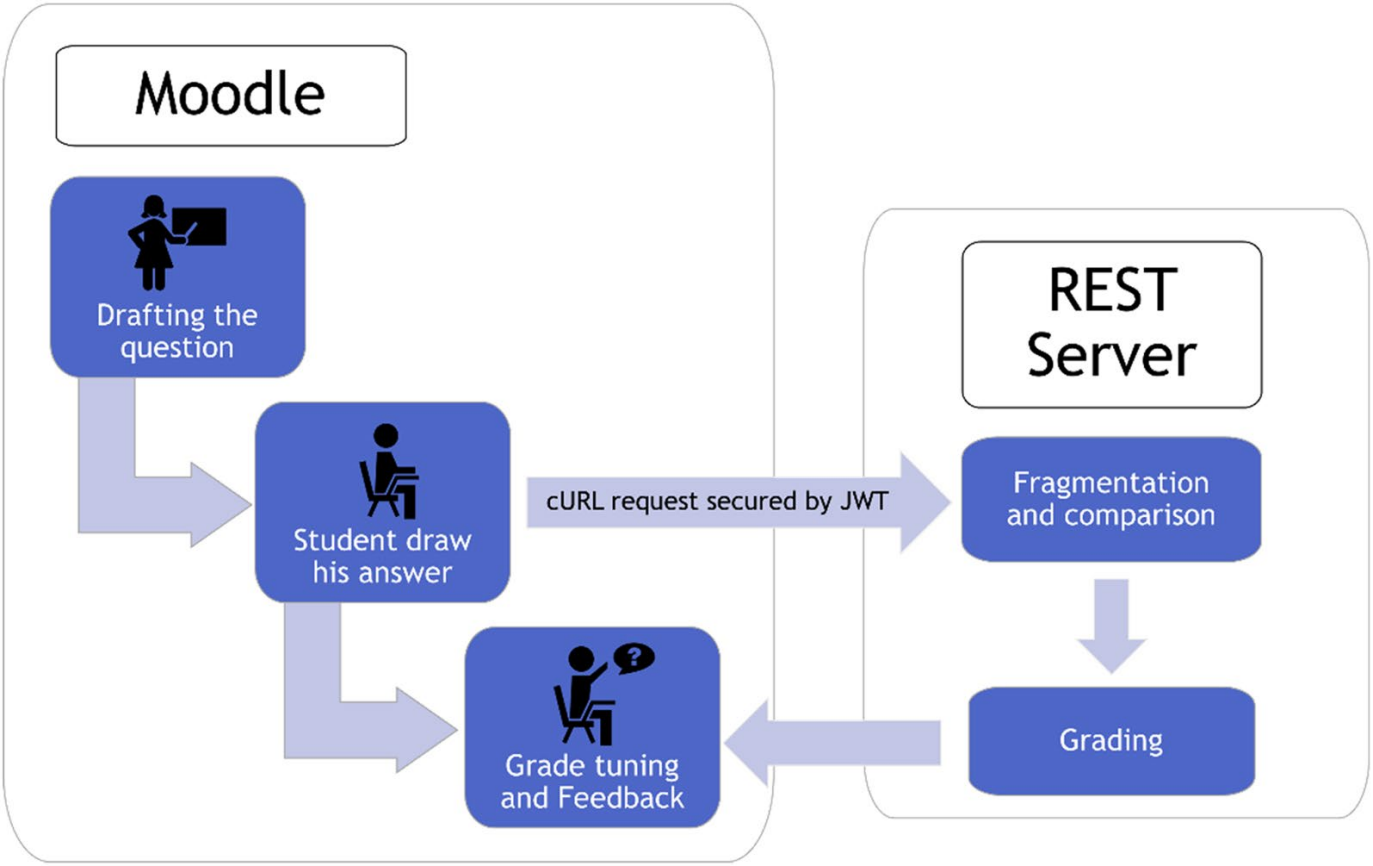
Workflow of the plugin. The teacher drafts a question, which is proposed to the student via the Moodle interface. The student’s and expected answers are sent to a server for comparison and soft grading. The grade is returned to Moodle for evaluation and feedback to the student

A workflow used by the developed plugin is shown in Fig. 1. In this implementation, we use an in-house correction algorithm hosted on a REST server. It computes the similarity between the student’s and teacher’s chemical structures. A REST server is, in our opinion, the most relevant technology in this context. It can be managed as suited by the end user–for instance, it can be installed on the same server as Moodle, encapsulated in a virtual machine or a different machine. The server uses little computing power. It doesn’t store any data and communicates exclusively with the Moodle server. Data is exchanged using the JSON (JavaScript Object Notation) [12] format, which is a standard in web applications and server transfers. The user interface is built using Chemdoodle Web Component [13], an open source JavaScript library providing a sketcher to draw chemical structures. It can export structures in both SDF (Structure Data File) and Chemdoodle JSON Format. The sketcher can also be used to import a MOL file instead of drawing the molecule. Some services of the sketcher, such as the support of other molecular file formats, have been disabled because they required connections to a foreign server. A pairwise Tanimoto similarity was computed using the ISIDA fragment descriptors [14, 15] generated with the help of the ISIDA Fragmentor2021 tool. ISIDA descriptors represent counts of subgraphs (fragments) of a molecular graph with defined topology and size, contrasting with fingerprint representations, in which a feature appears either present or absent.

A fragmentation scheme, or embedding, is defined by a set of parameters stored in the configuration file; thus, the administrator can tune the parameters if needed. The fragments used to compute the molecular similarity are enumerated from the chemical structure of the teacher’s answer; they are not pre-defined. The default fragmentation scheme supports organic chemical structures, eventually containing inorganic elements. It takes into account lone pairs, radicals, and formal charges. The grading is sensitive to the presence or absence of explicit hydrogens in the structures–the management of the hydrogens being a part of the evaluation.

The pairwise stereochemistry comparison is based on the InChI [16] strings generated with the help of the InChI v. 1.06 program [17]. The two compared structures without stereo labels must be identical. For them, the algorithm compares the information in related stereo-layers; for example, *[/t number_of_atom, stereo_label /m chirality_label*], where *stereo_label* = “ + ” and “−” and *chirality_label* = “0” and “1”.

The communication security between the Moodle server and the REST server is based on JWT (JSON Web Token) [18], an industry standard to secure requests between two entities. They contain three different parts: the header, the payload, and the signature. The header specifies the type of algorithm used to encrypt the signature and the type of token. The payload contains data, including the time at which the token has been issued. Both the header and the payload will be Base64Url encoded [19]. The signature is created using both the header, the payload, and a secret shared between Moodle and the REST API. Therefore, an attacker is not able to change the message without knowing the secret, as a given signature matches only one set of header, payload, and secret.

### Implementation

The plugin implementation involves three main steps: (i) formulating a question, (ii) answering a question, and (iii) displaying a teacher’s feedback. In this plugin, the teacher inputs from 1 to N deemed correct answers, allowing for several alternative structures (the plugin does not allow the teacher to define “inexact” answers). For instance, for the question “what is the structure of glucose?” both furanose and pyranose forms of glucose can be accepted as answers.

**Fig. 2:**
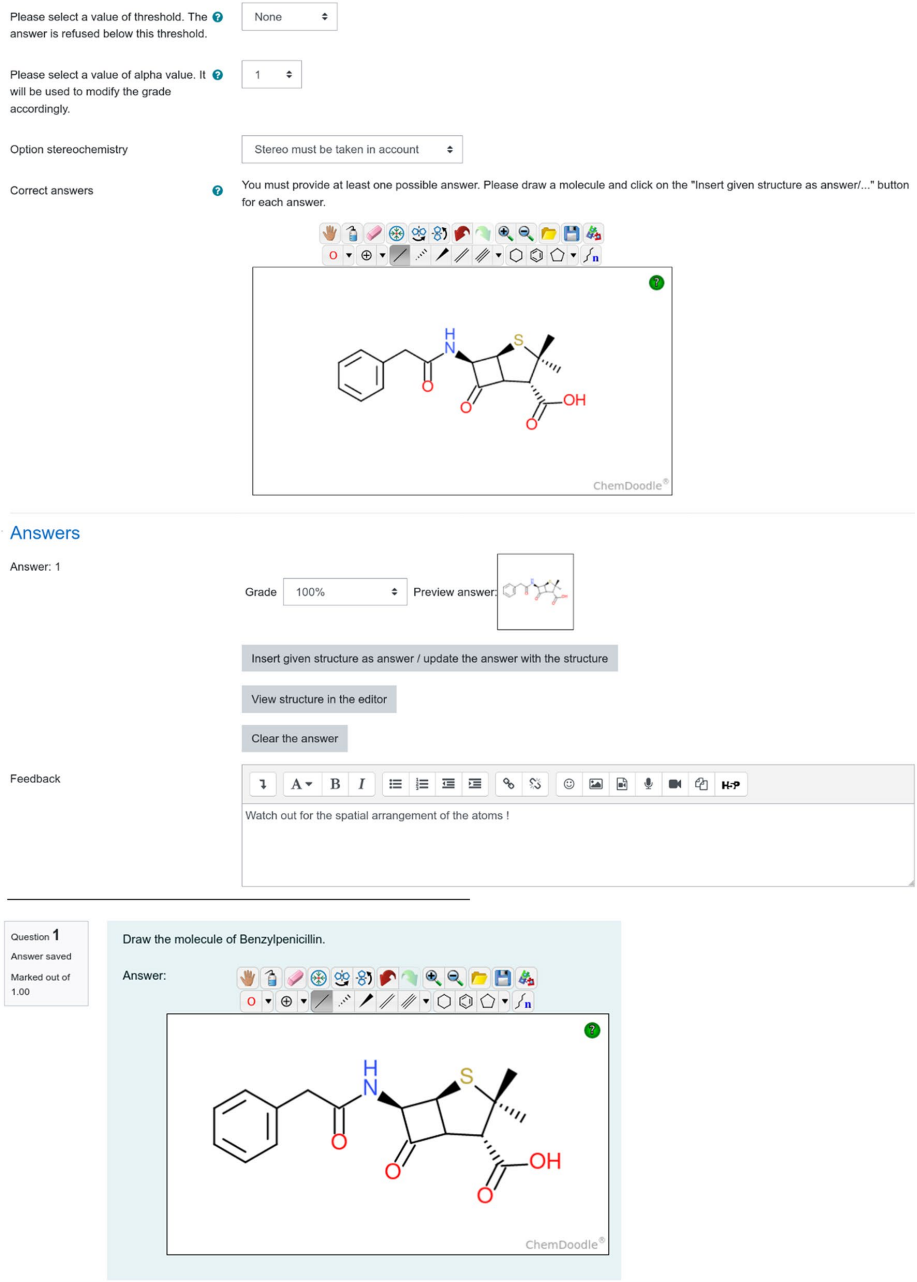
(Top) Drafting of the question by the teacher. The “Insert given structure as answer / update the answer with the structure” button inserts the current drawing as answer. The “View structure in the editor” button loads the data of the given answer to the sketcher. The “Clear the answer” button removes the answer. (bottom) Interface for the student to answer the question. The teacher’s instructions are displayed above the sketcher

Thus, for each answer, the teacher needs to draw the expected structure using the Chemdoodle Web Component Ketcher, then click on the ‘*Insert given structure as answer/update the answer with the structure’* button in order to insert this structure (Fig. 2, top). In this case, Chemdoodle JSON is used to encode a given chemical structure in MOL format. Both the Molfile and the Chemdoodle JSON will be saved as an answer in JSON format to the Moodle database.

Apart from chemical structures, the teacher can prepare instructions and feedback for the students. Two kinds of feedback are possible: “general” grade-unrelated feedback and “specific” feedback, displayed if a grade is inferior to 1, aiming to help students improve their answers. Upon taking a test, a student follows the teacher’s instructions in order to prepare the required chemical structure (Fig. 2, bottom).

Once submitted, the answer is processed using the same procedure as for the teacher’s question (see above). Then, the answers of both the teacher and the student are sent through a cURL [20] request to the correction REST API, written in the Pascal Object language [21]. Connections to the REST API are authenticated using the JWT standard. If the REST server does not respond, the Moodle administrator is notified, and the student’s answer is saved and marked as *Requires grading*. If a request is sent to the correction REST API and the authentication is not validated, the Moodle administrator is noticed that someone attempts to access to the correction REST API and receives related IP address.

Once the request is authenticated, the grade g_rest_ based on Tanimoto similarity between the student’s and teacher’s structures is computed on the REST server. Every chemical structure is encoded using the ISIDA molecular descriptors; by default, fragmentation IAB(2–4)FC_UR is used. It stands for sequences of 2–4 atoms and bonds, taking into account formal charges, lone pairs and radicals. It also includes atom count. If there are several structures prepared by the teacher, the highest Tanimoto score and the corresponding pair of student/teacher structures are kept for the upcoming steps. If the stereochemistry analysis is not requested, the grade g_rest_ is sent back to Moodle. Otherwise, InChI [16] strings are used to compare the teacher’s and student’s structures containing stereo-centers (the stereo centers can be either R/S or Z/E). The g_rest_ value is computed as the proportion of correctly drawn stereo centers (“$\#Correct Stereo Center$”) over the total number of stereo centers in the chemical structure (“$\#Total Stereo Center$”), and sent back to Moodle:

1 $${\text{g}}_{{{\text{rest}}}} = \left\{ \begin{gathered} \frac{{\# Correct~Stereo~Center}}{{\# Total~Stereo~Center}},~~if~~similarity~score~ = ~1 \hfill \\ 0,~~~~~~~~~~~~~~~~~~~~~~~~~~~~~~~\,\quad if~similarity~score~ \ne 1~ \hfill \\ \end{gathered} \right.$$

**Fig. 3:**
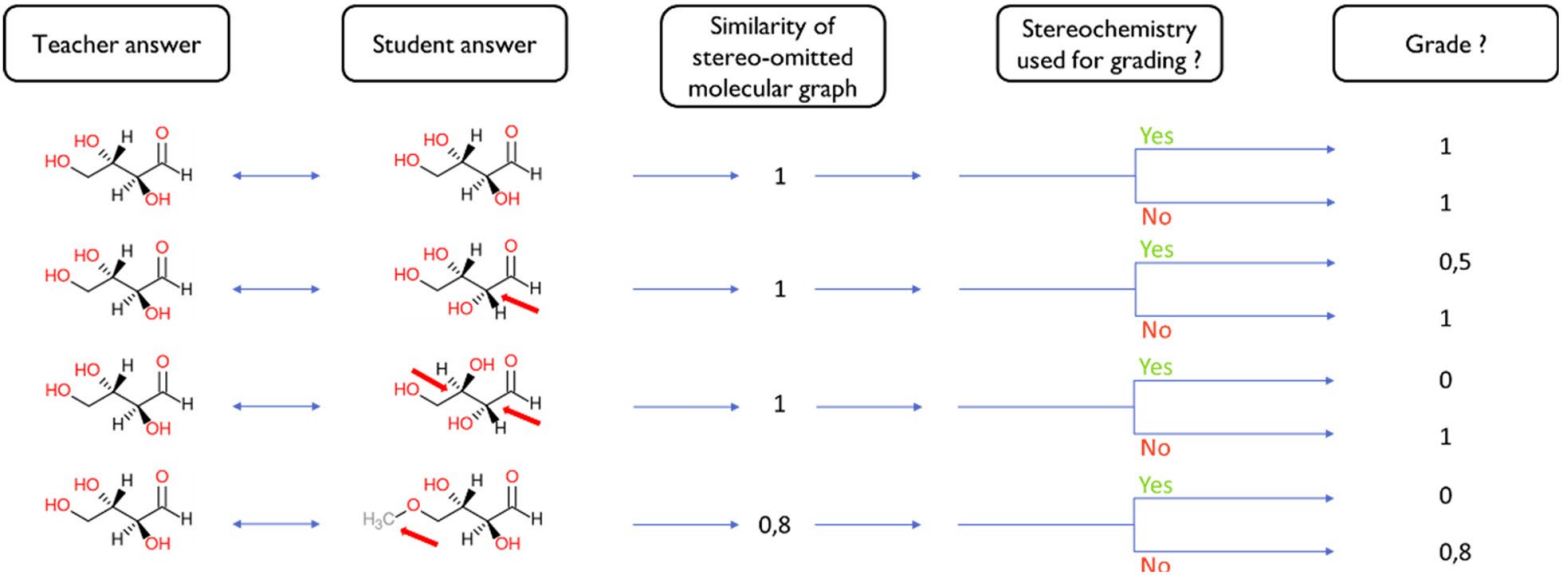
Illustration of the grading methodology. The red arrows are pointing at the differences between the teacher’s and the student’s answers. The chemical structures are compared by similarity first, to propose a grade. If the stereochemistry is evaluated, the proportion of correctly drawn centers is used to compute the grade

Notice that the stereocenter comparison becomes impossible if the structures (without stereo labels) are not identical. For this reason, if the similarity score is not equal to 1, a g_rest_ of 0 is returned to Moodle. Typical examples illustrating grading methodology including/excluding stereochemistry analysis are demonstrated in Fig. 3. For instance, if the student confuses an alcohol function with an ether, the Tanimoto similarity score student/teacher structures is 0.8. Therefore, the final grade is either zero, if the stereochemistry is required, or 0.8, otherwise.

Once the grade computed by the REST server is returned to the Moodle server, the final grade g is calculated according to formula (2) where t and α are user-defined parameters. Both parameters (α and t) can be set at the level of the question editor (Fig. 2, top) and can differ from question to question.

2 $$g = \left\{ \begin{gathered} \left( {g_{{rest}} } \right)^{\alpha } ,\quad if(g_{{rest}} )^{\alpha } \ge t \hfill \\ 0,\,other\,wise \hfill \\ \end{gathered} \right.$$

The parameter α modulates the teacher’s exigency: more lenient (α < 1) or more severe (α > 1). The t parameter is a threshold under which the grade is set to 0, to avoid attributing points to unacceptable answers.

**Fig. 4:**
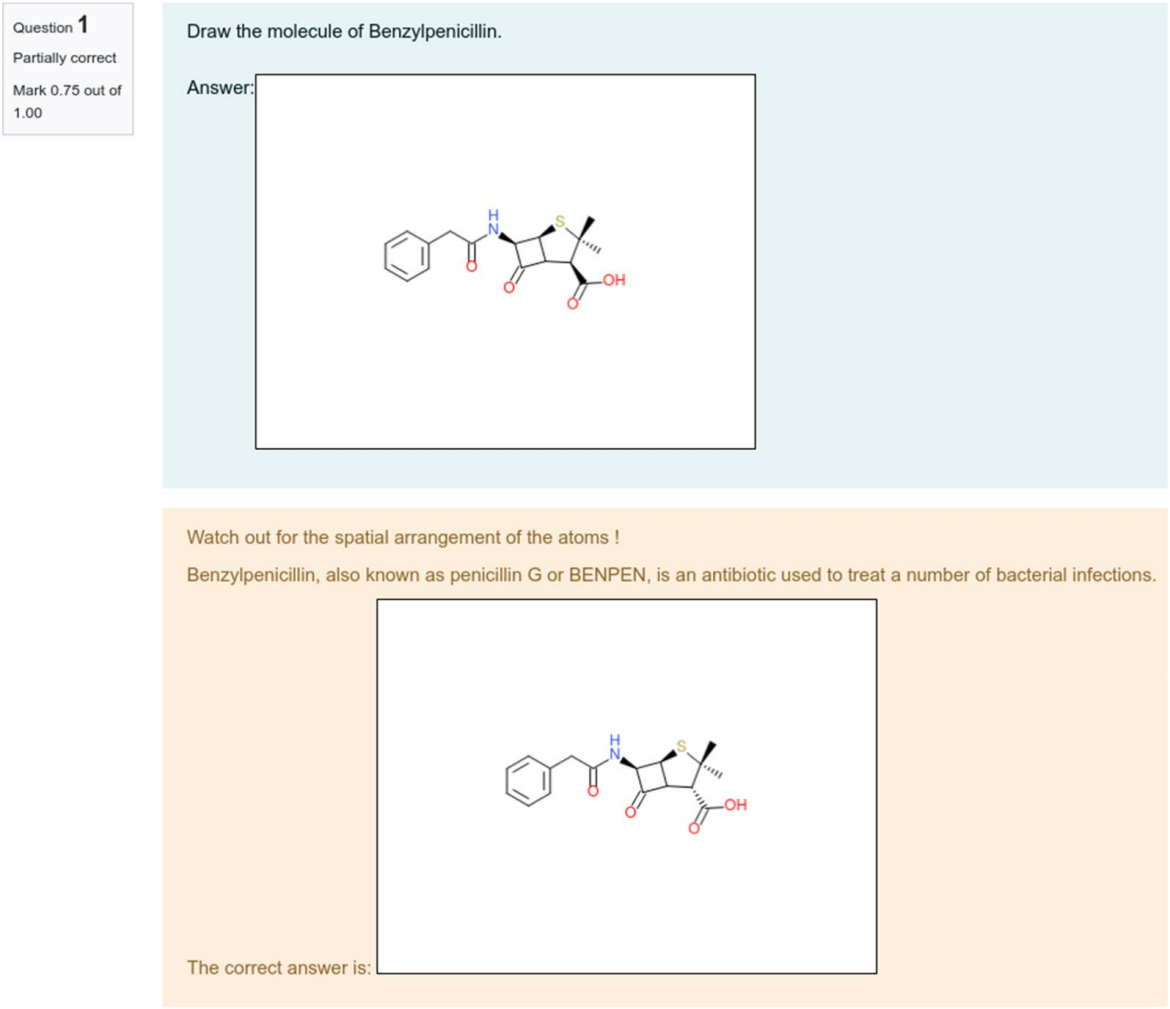
Interface of the feedback shown to the student once her/his answer has been corrected. Above is the answer of the student, below the expected answer with the teacher’s feedback. The mark of this question is displayed on the top left corner

Finally, the general feedback containing the expected answer is shown to the student, accompanied by the specific feedback if g < 1 (Fig. 4).

## Question examples

In this section, we describe several typical examples which can be realized with the developed plugin.

### Example 1. Drawing a Lewis structure

Both lone pairs, radicals and explicit/implicit hydrogens are considered. Since the correction is not binary, it allows students to be awarded some of the points even if some structural details are missed. For example, when asked for the Lewis structure of Nitrosyl Fluoride, forgetting one of the lone pairs on the Fluorine atom would result in a grade of 0.9/1.

### Example 2. Identification of the major product of a reaction

The soft grading system better assesses the student’s understanding of the regioselectivity, because the minor products is often similar with the major one. For example, if asked for the major product of 2,3-Dimethyl-2-butanol dehydration by H2SO4, the incorrect result would get a grade of 0.68.

### Example 3. Drawing a given configuration (R/S, E/Z) of a molecule

**Fig. 5:**
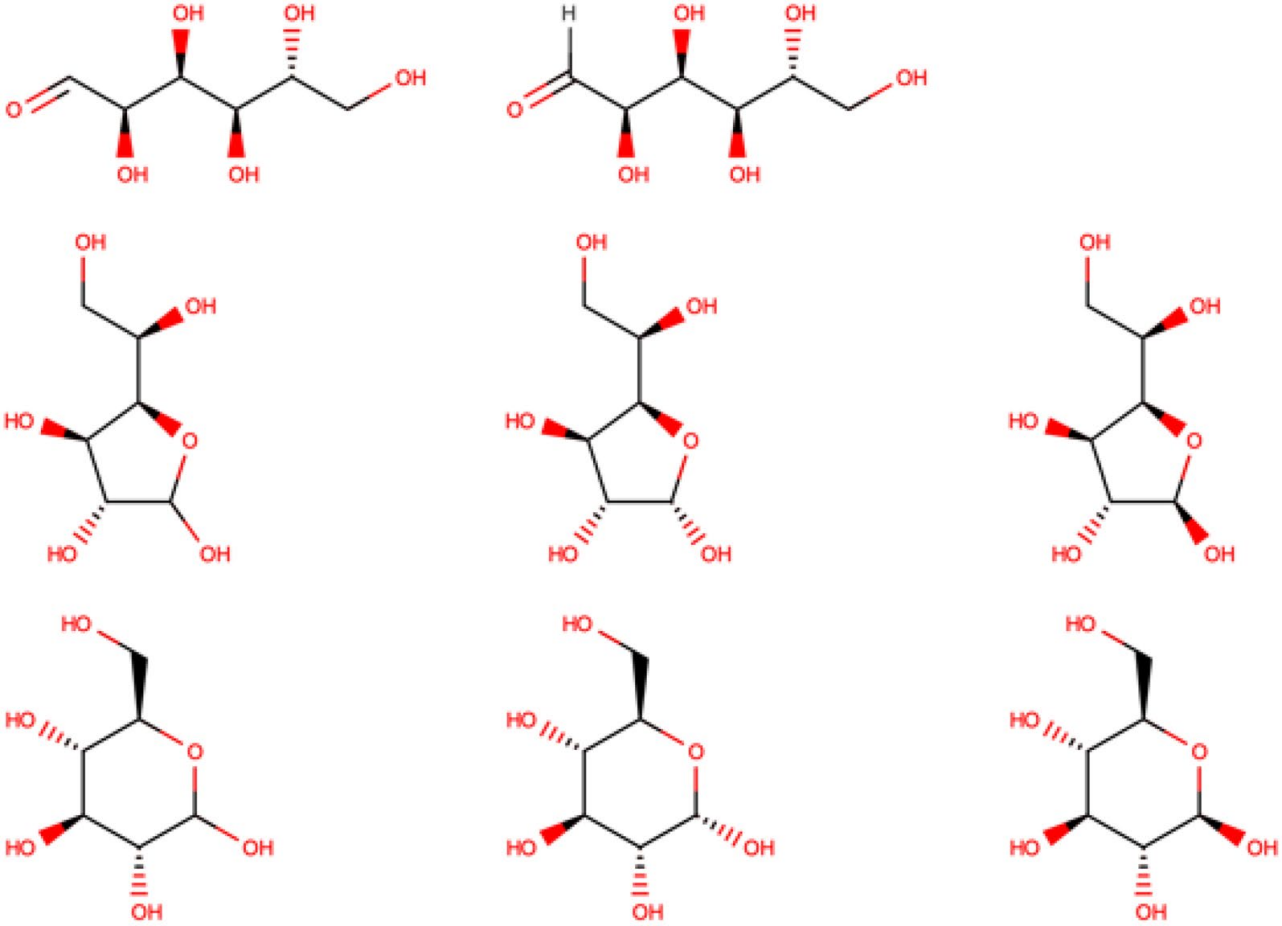
Eight possible answers expected by the teacher: 2 open structures (with and without explicit hydrogen); 3 furanoses and 3 pyranoses for the α-, β- and undefined isomers

**Fig. 6:**
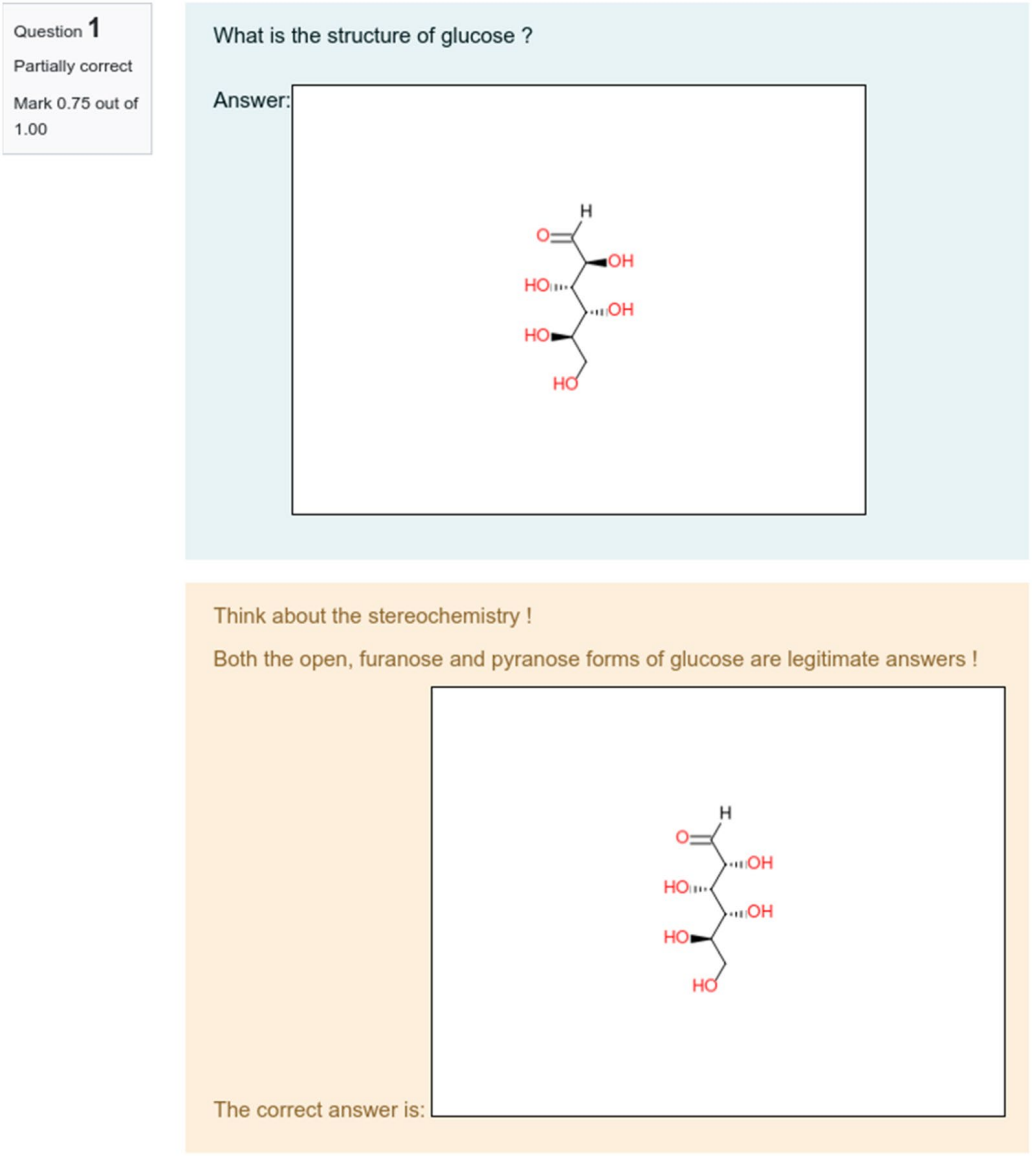
(top) The open form of glucose with explicit hydrogen on the carbonyl fragment and erroneous stereochemistry chosen by the student; (bottom) specific feedback and correct structure

If a compound has multiple stereo-centers but some stereo-centers were not found by the student, the soft grading system can be particularly useful. For instance, the question “what is the structure of glucose?” requires the student to consider stereochemistry. In this case, three structures are legitimate answers: open, furanose, and pyranose forms of glucose. Therefore, the teacher should prepare related structures by adding them as expected answers (see Fig. 5). Moreover, for the open form, the structures with both explicit and implicit hydrogen on the aldehyde group on the aldehyde need to be anticipated (Fig. 5, first line). Finally, the teacher needs to consider the stereo-orientation of the methoxy-substituent of the furanose and pyranose forms. In such a way, all 8 alternative structures of glucose (Fig. 5) must be considered as the correct answer. Let’s suggest that the student prepare the structure shown in Fig. 6, top. Compared to the closest teacher’s structure, s/he has correctly drawn 3 out of 4 stereo-centers. Thus, according to formula (2), his grade is 0.75. Both grade and teacher’s feedback are displayed after examination by the algorithm (Fig. 6, bottom).

### Comments

It should be noted that the soft grading is “global”, therefore it is presently not possible to give more importance to a given substructure.

## Conclusion

A new open source Moodle plugin for the assessment of chemical structures has been developed. It significantly extends an arsenal of chemical questions requiring students to draw chemical structures. A soft grading algorithm was implemented in order to reasonably assess the students’ skills. The tool is provided with the REST API server that can be used in any institution. It is highly secure with an authentication method needed to access the API, and it allows the teacher to ask chemical questions where the student has to draw her/his answer thanks to the implemented soft grading algorithm. This plugin only needs to be installed by the Moodle administrator of the institution, following the same procedure as any other Moodle plugin. It appears as a specific type of question when preparing a test. This work could be enhanced by the addition of several features, such as the possibility to consider wrong answers to give specific feedback to the students (for example, when requested to draw the major product of a reaction, it is desirable to consider the minor product as a wrong answer to provide some specific explanation to the student). The creation of a dedicated tool to automate the editing of questions using a set of chemical structures could be a useful addition–by generating questions in XML format that can be imported into Moodle, for instance. Other options to tune the soft grading are also desirable. For instance, it could be possible to let the teacher build the grade as a weighted sum of the structural similarity and the stereochemistry score. Another improvement could be to let the teacher decide if the module should standardize the protonation of the chemical structures. This work will be improved by the addition of a new layer which enables the asynchronous execution of the evaluation server, on dedicated Docker containers, managed by a RabbitMQ system (Queue message system) [22]. It is fully available from the git of the project: https://github.com/Laboratoire-de-Chemoinformatique/moodle-qtype_molsimilarity.

## Data Availability

Software is available on the web page of the project: https://github.com/Laboratoire-de-Chemoinformatique/moodle-qtype_molsimilarity. No additional data has been used. Project name: molsimilarity. Project home page: Git: https://github.com/Laboratoire-de-Chemoinformatique/moodle-qtype_molsimilarity Operating system(s): Linux, Mac & Windows. Programming language: PhP, JavaScript, Pascal Object. Other requirements: Moodle 3.9. License: GNU GPL v3 or later, IUPAC/InChI-Trust Licence No.1.0.
